# Stage specific requirement of platelet-derived growth factor receptor-α in embryonic development

**DOI:** 10.1371/journal.pone.0184473

**Published:** 2017-09-21

**Authors:** Chen Qian, Carol Wing Yan Wong, Zhongluan Wu, Qiuming He, Huimin Xia, Paul Kwong Hang Tam, Kenneth Kak Yuen Wong, Vincent Chi Hang Lui

**Affiliations:** 1 Department of Surgery, LKS Faculty of Medicine, The University of Hong Kong, Hong Kong SAR, China; 2 Department of Pediatric Surgery, Guangzhou Women and Children’s Medical Center, Guangzhou Medical University, Guangzhou, China; Academic Medical Centre, University of Amsterdam, NETHERLANDS

## Abstract

**Background:**

Platelet-derived growth factor receptor alpha (PDGFRα) is a cell-surface receptor tyrosine kinase for platelet-derived growth factors. Correct timing and level of *Pdgfra* expression is crucial for embryo development, and deletion of *Pdgfra* caused developmental defects of multiple endoderm and mesoderm derived structures, resulting in a complex phenotypes including orofacial cleft, spina bifida, rib deformities, and omphalocele in mice. However, it is not clear if deletion of *Pdgfra* at different embryonic stages differentially affects these structures.

**Purpose:**

To address the temporal requirement of *Pdgfra* in embryonic development.

**Methods:**

We have deleted the *Pdgfra* in *Pdgfra-*expressing tissues at different embryonic stages in mice, examined and quantified the developmental anomalies.

**Results:**

Current study showed that (i) conditional deletion of *Pdgfra* at different embryonic days (between E7.5 and E10.5) resulted in orofacial cleft, spina bifida, rib cage deformities, and omphalocele, and (ii) the day of *Pdgfra* deletion influenced the combinations, incidence and severities of these anomalies. Deletion of *Pdgfra* caused apoptosis of *Pdgfra*-expressing tissues, and developmental defects of their derivatives.

**Conclusion:**

Orofacial cleft, spina bifida and omphalocele are among the commonest skeletal and abdominal wall defects of newborns, but their genetic etiologies are largely unknown. The remarkable resemblance of our conditional *Pdgfra* knockout embryos to theses human congenital anomalies, suggesting that dysregulated *PDGFRA* expression could cause these anomalies in human. Future work should aim at defining (a) the regulatory elements for the expression of the human *PDGFRA* during embryonic development, and (b) if mutations / sequence variations of these regulatory elements cause these anomalies.

## Introduction

Platelet-derived growth factor (PDGF) was identified as a serum protein that stimulated the growth and migration of vascular smooth muscle cells, fibroblasts and glial cells [1–3]. In human and mouse, the PDGF family is composed of four different polypeptide chains (PDGF-A, -B, -C and -D) encoded by four different genes (*PDGF-A*, *-B*, *-C* and *-D*) [4–6]. PDGFs are secreted as homodimeric proteins (PDGF-AA, -BB, -CC and -DD) linked by two cysteine bridges. Only PDGF-A and PDGF-B are able to form heterodimer of PDGF-AB [6].

PDGFs bind to and signal through two cell-surface receptor tyrosine kinases, PDGFRα and PDGFRβ [7, 8]. PDGFRα has a broader specificity and bind PDGF-A, PDGF-B and PDGF-C, whereas PDGFR-β preferably binds PDGF-B and PDGF-D. PDGF-PDGFR signaling controls cellular proliferation, survival, migration and differentiation [9–11]. Gene knockout studies in mice have revealed critical roles of PDGFs and PDGFRs in embryo development.

PDGFRα is a transmembrane protein with an extracellular ligand binding domain and an intracellular tyrosine kinase domain, and functions as a typical receptor tyrosine kinase (RTK). PDGFRα binds homodimers of PDGF-A, PDGF-B, PDGF-C and heterodimer PDGF-AB [7, 8]. PDGFRα exists as monomeric form and PDGF binding dimerizes PDGFRαs, and activates the receptor's kinase activity. Tyrosine phosphorylation of the receptor itself and other substrates triggers intracellular signaling cascades, including Ras-MAPK (mitogen activated protein kinase), phosphatidylinositol 3-kinase (PI3K) and phospholipase C_ϒ_ pathways, which are essential for cellular proliferation, survival, migration and differentiation [10].

In mouse, *Pdgfra* was broadly expressed in primitive endoderm and mesoderm derivatives thorough embryogenesis, in that *Pdgfra* mRNA was detected in the visceral endoderm at E6.5, later after gastrulation, *Pdgfra* mRNA was detected in many areas of mesenchyme derivatives, including the somites, limb bud and branchial arches [12–14]. Deletion of *Pdgfra* induced embryonic lethality between E12 and E14, and caused defective development of many endoderm and mesoderm derived structures, resulting in cleft face, subepidermal blebbing, spina bifida, vertebrae and rib deformities, malformation of the shoulder girdle, hemorrhaging, and body wall musculature defect in *Pdgfra* null embryos [10, 11, 15]. In contrast, loss of *Pdgfrb* affected the development of a more restricted set of cell types, including the vascular smooth muscle cells such as the pericytes and the kidney mesangial cells, and that mice deficient of *Pdgfrb* displayed severe systemic vasculature defects and died shortly after birth [16, 17].

Sequence variants in the promoter and the 3’ untranslated region of *PDGFRA* gene have been identified to be associated with a number of human congenital developmental anomalies, which includes cleft palate [18], neural tube defects (spina bifida and anencephaly) [19–23], corneal astigmatism [24], and heart inflow tract defects [25], and some of these sequence variants affected the *PDGFRA* gene expression [18, 23]. Taken together with the results of *Pdgfra* knockout studies, it is clear that the correct time and level of expression of *Pdgfra* during embryonic development is crucial for proper development of many embryonic structures. However, it is not clear if deletion of *Pdgfra* at different embryonic stages differentially affects the development of these structures. To address the temporal requirement of *Pdgfra* in embryonic development, we have deleted the *Pdgfra* in *Pdgfra*-expressing tissues at different embryonic stages using a tamoxifen inducible *Cre-loxP* approach in mice, examined and quantified the developmental anomalies. Current study showed that conditional deletion of *Pdgfra* in *Pdgfra*-expressing tissues at different embryonic days (between E7.5 and E10.5) resulted in multiple developmental anomalies of the frontonasal region, the cranium, the vertebrae and the rib cage, and the abdominal wall in mice. Furthermore, the day at which *Pdgfra* was deleted had a major impact on the combination of the anomalies of the conditional *Pdgfra* knockout embryos.

## Materials & methods

### Mice and genotyping

The *Pdgfra*^*fl/fl*^ [15] and *Pdgfra*^*Cre/ERT*^ (*B6N;SJL-Tg(Pdgfr-a-cre/ERT)467Dbe/J*) [26] mouse strains were purchased from The Jackson Laboratory. A cDNA encoding tamoxifen-inducible Cre recombinase (*CreERT*) was inserted after the *Pdgfra* 5’UTR and followed by a rabbit *β*-globin poly A sequence in a mouse BAC clone (RP24-148N, BACPAC resource center, CHORI), that contained the mouse *Pdgfra* gene with 71kb upstream of the ATG and 41kb downstream of last coding exon. The BAC transgene was injected into pronuclei of one- or two-cell stage embryos of B6SJL mice to generate the *Pdgfra*^*Cre/ERT*^ transgenic mouse strain [26]. The *Pdgfra*^*fl/fl*^ mice were maintained in *C57BL/6JEi* and the *Pdgfra*^*Cre/ERT*^ mice were maintained in *C57BL/6NJ*.

All mice were supplied with food and water ad libitum, and kept under pathogen-free condition with a 12 h light/dark cycle. The morning the vaginal plug was observed was considered as embryonic day 0 (E0). All experiments were carried out in accordance with protocols approved by the Committee on the Use of Live Animals in Teaching & Research, The University of Hong Kong (CULATR No.: 3123–13, 3368–14; 3566–15).

### Genotyping

Genomic DNA was extracted from 2 mm tail clip using PBND extraction method (http://www.jax.org/imr/tail_nonorg.html). In brief, mouse-tail was digested with 40 mg Proteinase K (Invitrogen^™^) in 200 μl PBND buffer at 55°C for 16 hours. After heating at 96°C for 10 minutes to inactivate the Proteinase K, the tail digest was used as template DNA for PCR analysis. PCR reaction was performed in PCR buffer (25 μl) containing 0.2 mM dNTP (Promega), forward and reverse primer (0.2 μM each), template DNA (1 μl), DMSO (5%; v/v, Merck), MgCl_2_ (4 mM) and 0.25 μl of Ampli Taq GoldTM (Roche). Amplication was performed with forward and reverse primers specific for the *Cre*, wild-type and floxed *Pdgfra* and *Sry*. PCR products were resolved by running in a 1% (W/V) agarose gel (S1 Fig). Details of primers and sizes of their respective PCR products were shown in S1 Table.

To distinguish *Pdgfra*^*Cre/ERT*^; *Pdgfra*^*fl/fl*^ (CKO) and *Pdgfra*^*Cre/ERT*^; *Pdgfra*^*fl/+*^, we determined the copy number of *Pdgfra* by copy number assay with primers (*Copy Forward* & *Copy Reverse*) and TaqMan probe specific for the wild-type *Pdgfra* allele (S1 Fig). These primers and TaqMan probe will amplify and detect the wild-type *Pdgfra* and the *Pdgfra*^*Cre/ERT*^ allele but not the *Pdgfra*^*fl*^ allele. Using the mouse *Tfrc* (cat# 4458366) as an internal control for the copy number, the number of copy of *Pdgfra* allele were determined to distinguish *Pdgfra*^*Cre/ERT*^; *Pdgfra*^*fl/fl*^ (CKO) (*Pdgfra*^*Cre/ERT*^; *Pdgfra*^*fl/+*^ embryos (*Pdgfra*^*Cre/ERT*^; *Pdgfra*^*fl/fl*^ (CKO) has fewer copy of *Pdgfra* than *Pdgfra*^*Cre/ERT*^; *Pdgfra*^*fl/+*^). Copy number assays were performed following TaqMan^®^ Copy Number Assay Protocol (Applied Biosystem). Briefly, the 20 μl assay mixture contained 2×TaqMan universal PCR Master Mix (Applied Biosystems), 1 μl of each primer, and 40 ng DNA Template. DNA was amplified in ABI 7900HT Fast Real-Time PCR System (Applied Biosystems). The copy number of *Pdgfra* was calculated using *Tfrc* as internal control using SDS software version 2.0 (Applied Biosystems). All samples were run in quadruple with negative and positive controls. The median value of the quadruple triplicate results was recorded.

### Tamoxifen induction

Tamoxifen stock (20 mg/ml of corn oil) was prepared by warming tamoxifen (Sigma) in corn oil at 65°C (protected from light) until completely dissolved. Tamoxifen (0.1 mg per gram of body weight) was administered by intragastric gavage to pregnant mice.

### Morphology and histology examinations

Embryos were harvested for morphology examination for anomalies. Photos were taken with the Olympus SZX7 microscope mounted with the Olympus DP71 High Resolution Color Digital Camera.

For histology, embryos were fixed in PFA/PBS (4% (w/v) paraformaldehyde (Sigma-Aldrich, Steinheim, Germany) in PBS (phosphate-buffered saline, pH 7.2; Sigma-Aldrich, Steinheim, Germany)) for 18 hours at 4°C, dehydrated in graded series of alcohol (Merck, Darmstadt, Germany), cleared in xylene (RCI Labscan Ltd, Bangkok, Thailand) before being embedded in paraffin (Leica Biosystems, Richmond, IL USA). Sections (8 μm in thickness) were prepared, mounted onto TESPA-coated microscope glass, and stained with hematoxylin and eosin following standard protocol. Photos were taken with the Olympus SZX7 microscope mounted with the Olympus DP71 High Resolution Color Digital Camera.

### Skeletal staining

Skeletons of mouse embryos were stained with alizarin red S and alcian blue following the protocol as published previously [27]. In brief, embryos were eviscerated and then fixed in 100% ethanol (Merck, Darmstadt, Germany) for 4 days followed with acetone for 3 days. After fixation, embryos were rinsed with water, and then incubated in staining solution containing alizarin red S (0.06%; w/v; Sigma-Aldrich, Steinheim, Germany), alcian blue 8GX (0.02%; w/v; Sigma-Aldrich, Steinheim, Germany), 5% glacial acetic acid in ethanol for 10 days. After staining, the embryos were rinsed with water, then incubated in 20% glycerol (v/v; Sigma-Aldrich, Steinheim, Germany), 1% KOH (w/v; BDH, Poole England) at 37°C for 8 hours, and then incubated in the same solution at room temperature until tissue was completely cleared. Then the solution was replaced with 50% glycerol, 80% glycerol and finally 100% glycerol (USB Corporation, Cleveland, OH USA). Photos were taken with the Olympus SZX7 microscope mounted with the Olympus DP71 High Resolution Color Digital Camera.

### Whole mount beta-galactosidase activity staining

Embryos were fixed in PFA/PBS for 1 hour at 4°C. X-gal/IPTG staining was performed at 37°C for 18 hours as describe previously [28]. Stained embryos were fixed in PFA/PBS at 4°C for 18 hours, dehydrated in graded series of alcohol, cleared in toluene (RCI Labscan Ltd, Bangkok, Thailand) before being embedded in paraffin. 10-μm-thick sections were prepared and mounted onto TESPA-coated microscope glasses. Sections were dewaxed in toluene, hydrated in in graded series of alcohol before being completely hydrated in water, counter-stained with Nuclear Fast Red according to manufacturer’s protocol (Vector Lab. Inc. Burlingham, CA), and mounted in Faramount Aqueous Mounting Medium (Dako).

### Immuno-histochemistry

Paraffin sections (8μm in thickness) were prepared and mounted onto TESPA-coated glass slides. Sections were dewaxed in xylene, hydrated in a graded series of alcohol and finally in distilled water. Endogenous peroxidase activity was quenched by incubation of section in methanol containing 3% H_2_O_2_ at room temperature for 30 minutes. After washing in water, antigen was retrieved by incubating in 10 mM sodium citrate buffer (pH 6.0) at 95°C for 10 minutes. After blocking in PBS-T (PBS with 0.1% Triton) supplemented with 1% Bovine Serum Albumin (USB Corporation, Cleveland, OH USA) for 1 hour at room temperature, sections were incubated with anti-PDGFRA antibody (1:200; ab21286; Abcam) in PBS-T for overnight at 4°C. The sections were washed in PBS-T and secondary body incubation and signal development were performed using EnVision Detection Systems Peroxidase/DAB, Rabbit/Mouse system according to manufacturer’s protocol (Dako). After washing in water, sections were counterstained with Haematoxylin, washed in water, incubated in 2% (W/V) NaHCO_3_ for 30 seconds before being washed in water. Sections were dehydrated in a graded series of alcohol, dewaxed in xylene and then mounted in DPX mountant (BDH). Images were taken with Nikon Eclipse E600 microscope mounted with Nikon Digital Camera DXM1200F.

### Von Kossa staining

Paraffin sections (8 μm in thickness) were prepared and mounted onto TESPA-coated glass slides. Sections were dewaxed in xylene, hydrated in a graded series of alcohol and finally in distilled water. Sections were incubated with 1% AgNO_3_ (W/V) for 45 minutes under strong light (60 watt tungsten lamp), washed in distilled water, then incubated with 3% (W/V) Na_2_S_2_O_3_ for 5 minutes before being washed in distilled water. Sections were counterstained with Haematoxylin, washed in water, incubated in 2% (W/V) NaHCO_3_ for 30 seconds before being washed in water. Sections were dehydrated in a graded series of alcohol, dewaxed in xylene and then mounted in DPX mountant (BDH). Images were taken with Nikon Eclipse E600 microscope mounted with Nikon Digital Camera DXM1200F.

### TUNEL assay

Apoptotic cells on sections were detected using *In Situ* Cell Death Detection Kit following manufacturer’s protocol (Roche Applied Science, Indianapolis, IN, USA). Sections were mounted in VECTORSHIELD^®^ mounting medium with DAPI (Vector Lab. Inc., Burlingame, CA 94010). Images were taken with Nikon Eclipse 80i microscope (Melville, NY, USA) mounted with SPOT RT3 microscope digital camera (DIAGNOSTIC Instruments, Inc., Sterling Heights, MI, USA) under fluorescence illumination for the DAPI+ve nuclei and TUNEL+ve cells. Photos were compiled using Adobe Photoshop 7.

To quantitate the apoptosis of the CKO embryos and the control embryos, photos of the TUNEL stained embryo sections were taken under x100 magnification, total number of cells of the branchial arch, somite and the abdominal wall were quantitated by counting DAPI+ve nuclei and apoptotic cells were quantitated by counting TUNEL+ve nuclei. Percentages of apoptotic cells of the respective regions of CKO and control embryos were calculated by (TUNEL+ve nuclei/DAPI+ve nuclei) x100%. Four to five embryos of each group were included for the analysis, three sections of each embryos were examined for the quantitation of the apoptosis. Percentages of apoptosis were shown as mean±S.D.

### Statistical analysis

Student's t Test was performed to calculate the differences between groups, and *p* value less than 0.05 was regarded as statistically significant.

## Results

### Tamoxifen inducible cre-mediated deletion of gene in *Pdgfra*-expressing tissues in mouse

**Fig 1:**
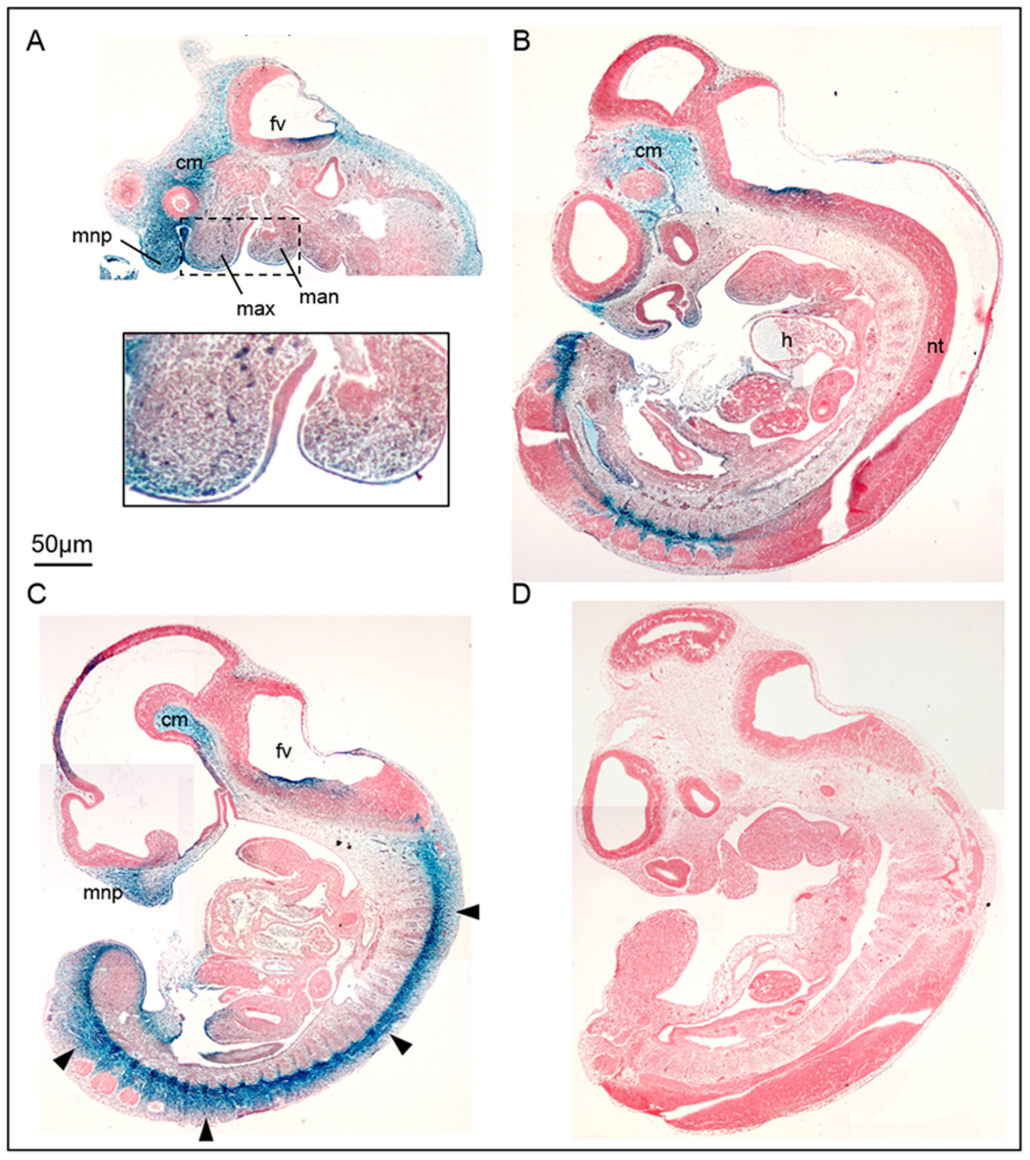
*Pdgfra*^*Cre/ERT*^ induced robust Cre-mediated recombination in *Pdgfra*-expressing tissues in mouse embryos. We crossed the *Pdgfra*^*Cre/ERT*^ mice to *Rosa26R* (*R26R*) and tamoxifen (Tm) was administered at E9.5 by intragastic gavage. Embryos were collected at E11.5 and processed for whole mount X-gal staining. Sagittal sectioning of the X-gal stained double transgenic (A-C) and control (D) (*Pdgfra*^*Cre/ERT*^ or *R26R*) littermates were shown for comparison. Highlighted regions were magnified and shown as insets. Abbreviations: fv, forth ventricle; cm, cephalic mesenchyme; mnp, medial nasal process; max, maxillary process; man, mandibular process; h, heart; nt, neural tube.

To address the temporal requirement of *Pdgfra* in embryonic development, we employed *Pdgfra*^*Cre/ERT*^ transgenic mouse [26] to delete the *Pdgfra* gene at different embryonic days by tamoxifen inducible *Cre-loxP* approach in mice, examined and quantified the developmental anomalies of the *Pdgfra* conditional knockout mutants. The *Pdgfra*^*Cre/ERT*^ transgenic mouse line directed tamoxifen inducible Cre recombination in *Pdgfra* expressing oligonucleotides [26], but the expression of the tamoxifen inducible Cre recombinase activity in other *Pdgfra* expressing tissues has not been reported. First, we crossed the *Pdgfra*^*Cre/ERT*^ mice to *Rosa26R* (*R26R*) [29] and tested if tamoxifen induced a deletion of stuffer of the *R26R* loci and X-gal expression to the regions where the endogenous *Pdgfra* was expressed in mouse embryos. Tamoxifen (Tm) administration at E9.5 induced a robust X-gal expression to the mesoderm of the cephalic region, the medial nasal process, the maxillary and mandibular processes, the developing somites of the E11.5 embryos (Fig 1). Furthermore, X-gal expression could already be detected in *Pdgfra*^*Cre/ERT*^; *R26R* embryos as early as 24 hours after Tm administration at E9.5 (Data not shown).

### Conditional knockout of *Pdgfra* in *Pdgfra*-expressing tissues in early embryonic periods resulted in embryonic lethality

We crossed the *Pdgfra*^*Cre/ERT*^; *Pdgfra*^*fl/+*^ mice with *Pdgfra*^*fl/fl*^ mice and gave single dose of Tm to the pregnant mice at E7.5, no CKO embryos could be collected at E18.5, which indicated that knockout of *Pdgfra* at early embryonic day E7.5 was embryonic lethal, which was in line with embryonic lethality of *Pdgfra*^*-/-*^ mutant mice [11, 15]. Male and female E14.5 CKO embryos of the E7.5 Tm treatment groups could be recovered in compliance with the expected Mendelian inheritance, and all the E14.5 CKO embryos of the E7.5 Tm groups displayed various degree of facial cleft, which could be attributable to the variation of cre-mediated knockout efficiency of *Pdgfra* in embryos. In addition, growth retardation, subepidermal blebs (arrowhead) and extensive hemorrphaging were also observed in mutant embryos (S2 Fig). Similar developmental defects and embryonic lethality were also observed in CKO embryos when Tm was given at E8.5 (Data not shown). The phenotypes of CKO embryos were identical to those of *Pdgfra*^*-/-*^ mutant mice [11, 15], which indicated that the *Pdgfra*^*Cre/ERT*^ transgenic mouse [26] directed robust and specific expression of Cre to most of the tissues that express the endogenous *Pdgfra* gene.

### Conditional knockout of *Pdgfra* in *Pdgfra*-expressing tissues in embryos resulted in axial skeletal and body wall developmental anomalies

**Fig 2:**
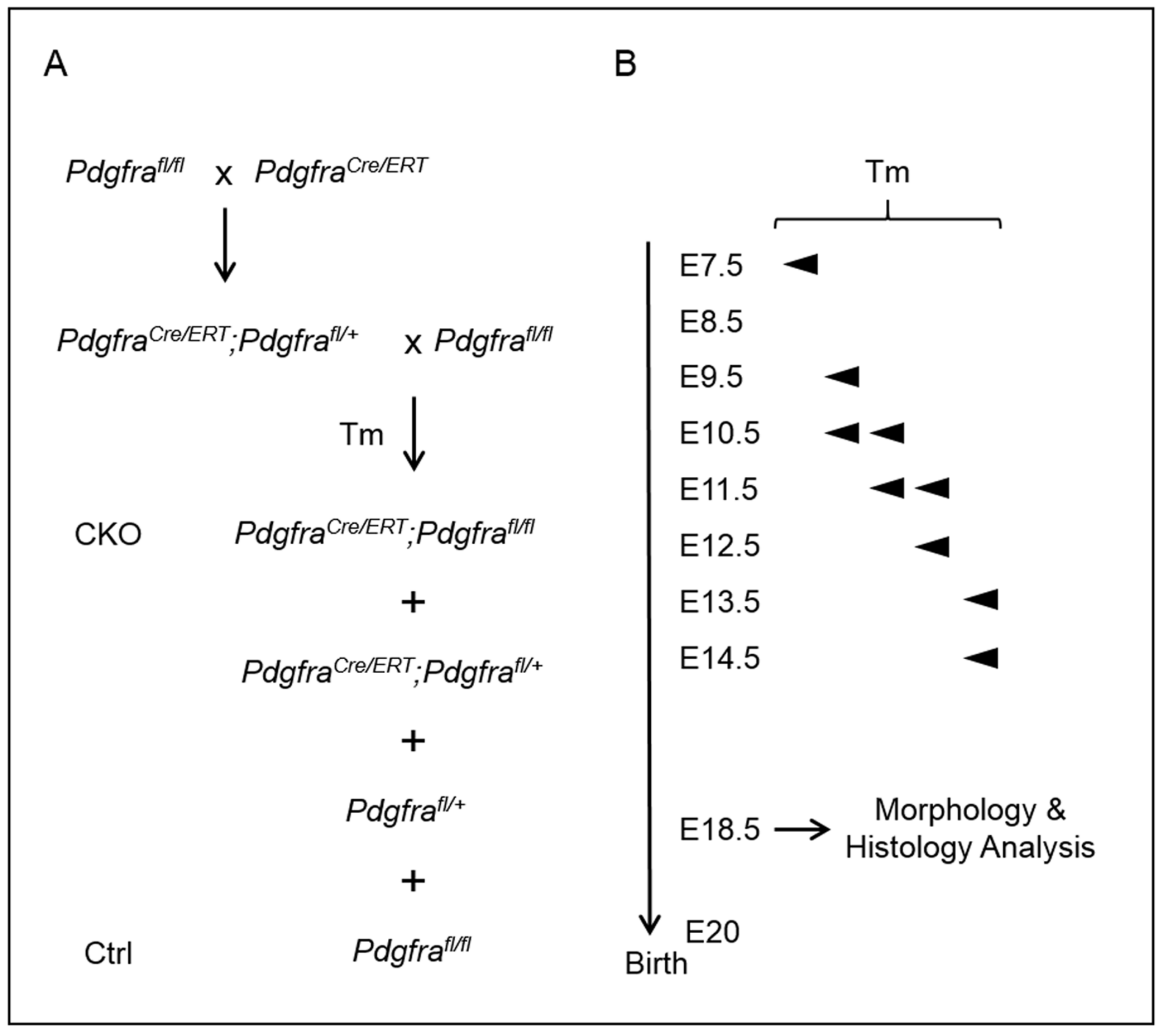
Conditional deletion of *Pdgfra* in *Pdgfra*-expressing tissues in mouse embryos. (A) Crossing scheme of *Pdgfra*^*Cre/ERT*^ mice with *Pdgfra*^*fl/fl*^ to generate conditional knockout (CKO, *Pdgfra*^*Cre/ERT*^;*Pdgfra*^*fl/fl*^) and control (Ctrl, *Pdgfra*^*fl/fl*^) embryos. (B) Tamoxifen (Tm) was given to pregnant females at various embryo days by intragastic gavage, and embryos were collected for the analysis.

The early embryonic lethality of CKOs of E7.5 and E8.5 Tm groups precluded the study of the impact of the conditional knockout of *Pdgfra* in the axial skeleton and body wall development beyond E14.5. To circumvent the problem of embryonic lethality and to investigate the impact of the conditional knockout of *Pdgfra* in *Pdgfra*-expressing tissues on the axial skeletal and body wall development, we gave Tm to pregnant females for two consecutive days starting at a slightly later embryonic days of E9.5, E10.5, E11.5 and E13.5, harvested embryos at E18.5 for examination (Fig 2). Male and female E18.5 CKO embryos of all these Tm treatment groups were recovered in compliance with the expected Mendelian inheritance (Table 1). Immuno-histochemistry for PDGFRA on sections showed that Tm administration at E9.5 markedly reduced the expression level of PDGFRα in E12.5 CKO embryos (S3 Fig).

**Table 1:** Phenotypes of *Pdgfra* CKO embryos of different Tm groups.

Tm	No. of embryo (n)	Mutant (n)	Cleft face	Short snout	Cranium defect	Cleft palate	Omphalocele	Spina bifida
E9.5	180	48	11 (23%)	26 (54%)	37 (77%)	11 (23%)	48 (100%)	31 (65%)
E10.5	45	12	0 (0%)	1 (8%)	1 (8%)	0 (0%)	12 (100%)	2 (17%)
E11.5	23	5	0 (0%)	0 (0%)	0 (0%)	0 (0%)	5 (100%)	0 (0%)
E13.5	10	3	0 (0%)	0 (0%)	0 (0%)	0 (0%)	0 (0%)	0 (0%)

% of the CKO embryos showing the respective anomaly was shown in parenthesis

**Fig 3:**
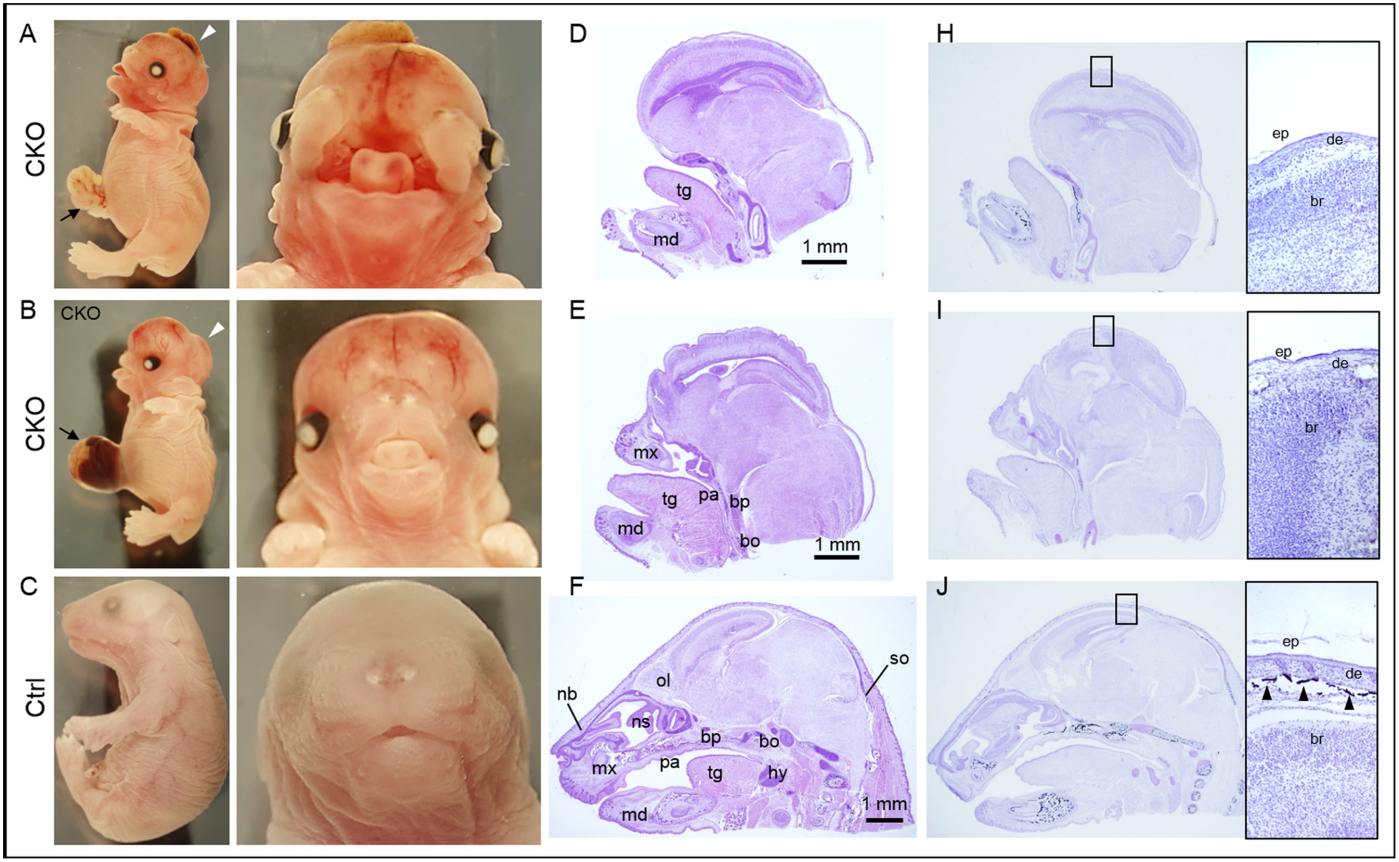
Conditional knockout of *Pdgfra* in *Pdgfra*-expressing tissues in embryos at E9.5 resulted in multiple developmental anomalies. *Pdgfra*^*Cre/ERT*^;*Pdgfra*^*fl/+*^ mice were crossed with *Pdgfra*^*fl/fl*^, tamoxifen was given to pregnant females at E9.5 for two consecutive days. Conditional knockout (CKO, *Pdgfra*^*Cre/ERT*^;*Pdgfra*^*fl/fl*^) and control (Ctrl, *Pdgfra*^*fl/fl*^) embryos were collected at E18.5 for analysis. Morphological examinations of CKO embryos (A, B) and control littermates (C). Haematoxylin and eosin staining of sagittal sections of the head of CKO embryos (D, E) and control littermates (F). Von Kossa staining (black) of the sagittal sections of the head of CKO embryos (H, I) and control littermate (J). Highlighted regions were magnified and shown on the right, and arrowheads indicated the positive staining of bone. Arrowheads indicated severe midline frontonasal clefting with exencephaly, and arrows indicated omphalocele. Abbreviations: tg, tongue; md, mandible; mx, maxilla; pa, palatal shelf; bp, basisphenoid bone; bo, basioccipital bone; nb, nasal bone; ns, nasal septum; hy, hyoid bone; so, supraoccipital bone; ep, epidermis; de, dermis; br, brain; bo, bone.

**Fig 4:**
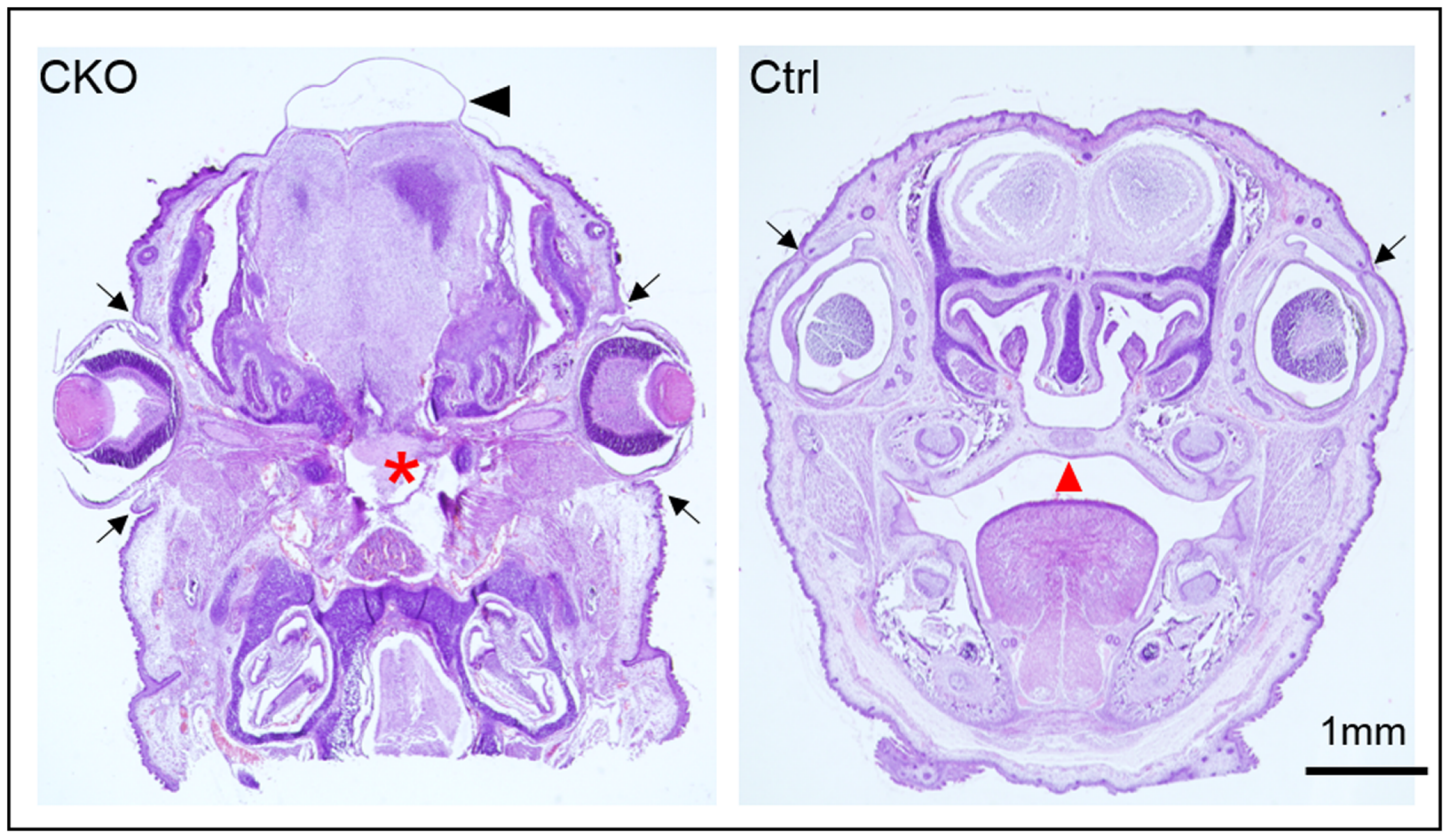
Craniofacial anomalies of conditional *Pdgfra* knockout embryos. Coronal sections of the head of conditional knockout (CKO, *Pdgfra*^*Cre/ERT*^;*Pdgfra*^*fl/fl*^) and control (Ctrl, *Pdgfra*^*fl/fl*^) E18.5 embryos were processed for haematoxylin and eosin staining. In CKO embryos with cleft face, the nasal capsule failed to fuse, and the palatal shelf (asterisk) failed to form. The cranium failed to develop, the brain was covered with a thin skin and edema (arrowhead) was formed. Arrows indicated the eyelids.

From the E9.5 Tm group, 23% of CKO embryos (11 out of 48 CKO embryos) displayed a severe midline frontal nasal clefting spanning the anterior forebrain to the frontal nasal process (Fig 3A). Although facial cleft was not observed in all the CKO embryos, over half of the other CKO embryos (26 out of 48 CKO embryos, 54%) showed markedly shorter snout with tongue protrusion (Fig 3B). Histological examination revealed a lack of or retardation of skeletal development of the frontal nasal regions in these CKO embryos (Fig 3D and 3E). In the CKO embryos with cleft face, the nasal capsule with the frontal and nasal bones were lacking or failed to fuse (Fig 3D). In those CKO embryos with short snout, the nasal capsule was severely developmentally retarded and the nasal septum was lacking (Fig 3E). The palatal shelves failed to form resulting in cleft palate in all the CKO embryos with cleft face, in that the brain communicated directly with the nasal and the oral cavities (Fig 4; Table 1).

All the CKO embryos of E9.5 Tm group with either cleft face or short snout (37 out of 48 CKO embryos, 77%) displayed cranial skeletal defect with the entire skull bone missing, and the brain was covered only with the skin (Fig 3A and 3B). Exencephaly, a type of cephalic disorder wherein the brain was located outside of the skull, was observed in 75% of the CKO embryos with cleft face and in 50% of the CKO embryos with short snout (arrowheads; Fig 3A and 3B). The absence of skull bone in these CKO embryos was further confirmed by Von Kossa staining of the cranial regions (Fig 3D–3F). The neural crest derived basisphenoid bone, the cephalic mesoderm derived bones including the supraoccipital and basioccipital bones were either missing or retarded in the CKO embryos with cleft face or short snout (Fig 3). The eyes were covered with eyelids in control littermates but they were not covered in CKO embryos (Fig 3). The upper and lower eyelids of the left and right eyes of control (arrows) have completely grown covering the cornea in controls. In contrast, the upper and lower eyelids (arrows) were not developed in CKO embryos (Fig 4).

**Fig 5:**
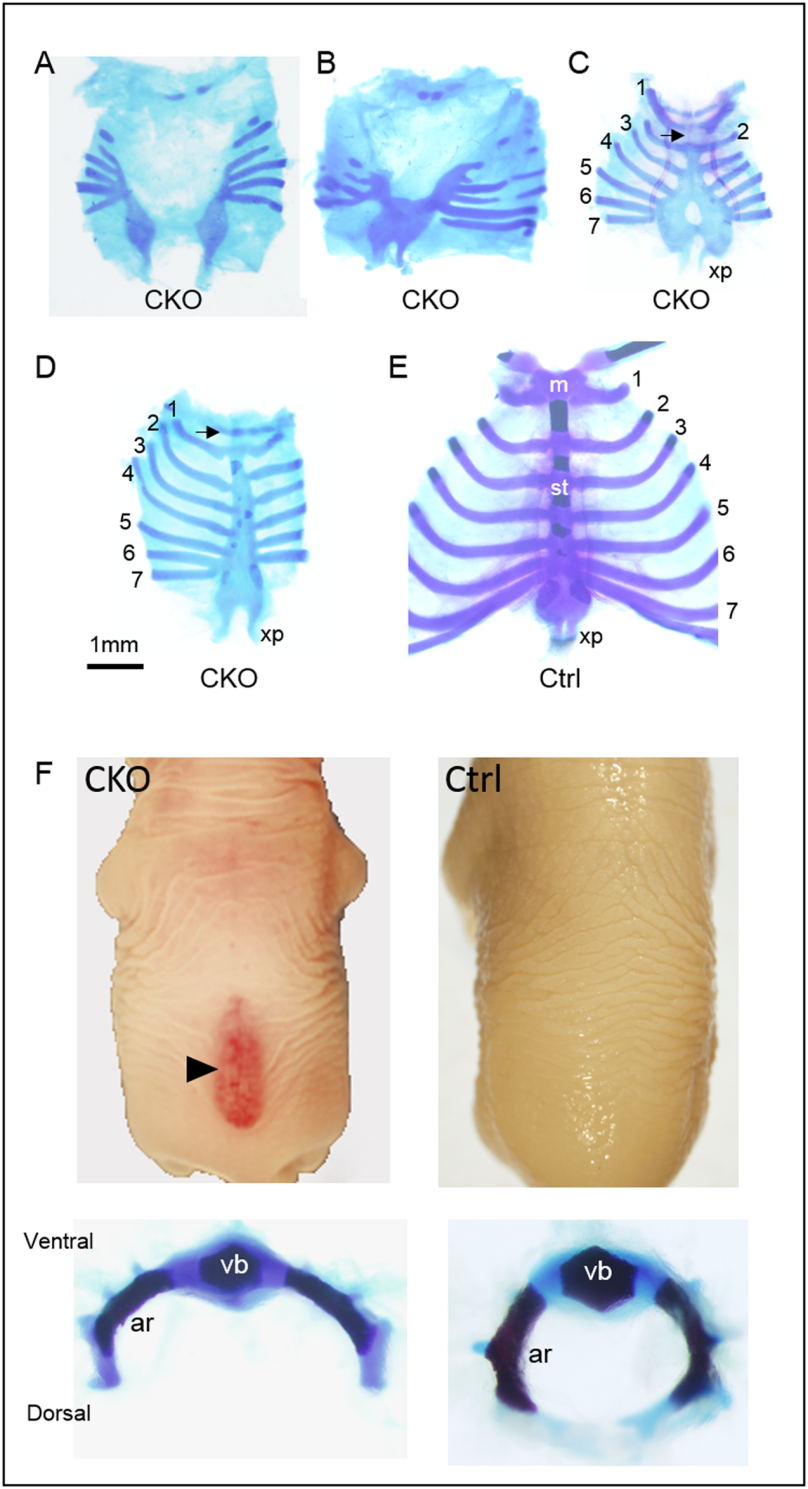
Axial skeletal anomalies of conditional *Pdgfra* knockout embryos. Skeletal staining of the rib cages of conditional *Pdgfra* knockout (CKO, *Pdgfra*^*Cre/ERT*^;*Pdgfra*^*fl/fl*^) (A-D) and control (Ctrl, *Pdgfra*^*fl/fl*^) (E) embryos were shown for comparison. The ribs were numbered and arrows indicated the respective location of the manubrium. CKO embryos developed spina bifida (arrowhead), and the vertebral arches of the CKO vertebrae failed to form the convex structure as observed in the control littermate (F). Abbreviations: m, manubrium; st, sternum; xp, xiphoid process; vb, vertebral body; ar, vertebral arch.

**Fig 6:**
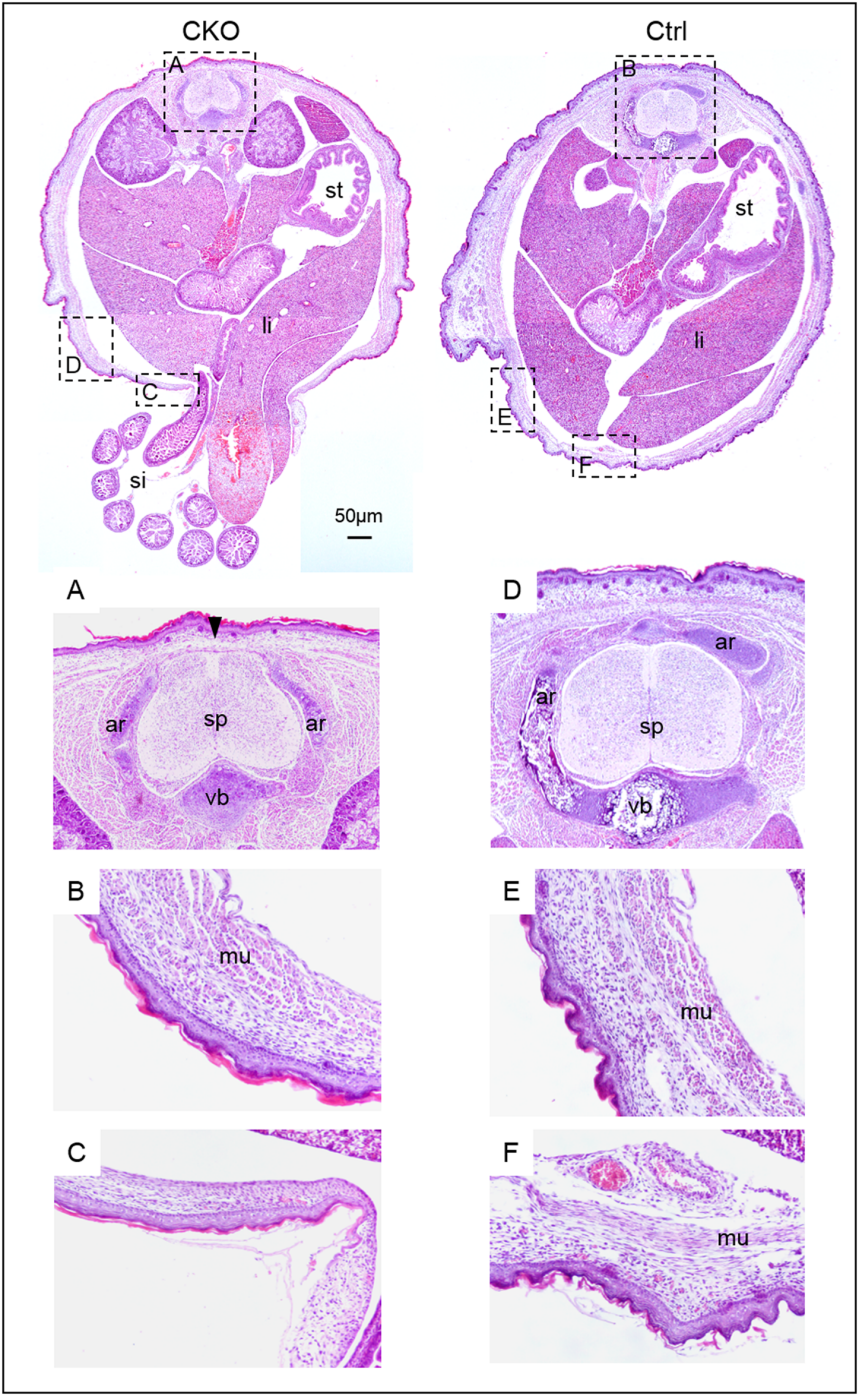
Ventral body wall muscle defects of conditional *Pdgfra* knockout embryos. Haematoxylin and eosin staining of transverse sections of the trunk region of CKO (*Pdgfra*^*Cre/ERT*^;*Pdgfra*^*fl/fl*^) embryos and control (*Pdgfra*^*fl/fl*^) littermate (Ctrl) were shown for comparison. Highlighted regions were magnified and shown. Abbreviations: st, stomach; li, liver; si, small intestine; sp, spinal cord; vb, vertebral body; ar, vertebral arch; mu, muscle.

Skeletal abnormalities were also detected in the vertebral column and the rib cavity of the CKO embryos of the E9.5 Tm group. All the CKO embryos displayed rib cage abnormalities of various severities. In generally, the sternum was shorter and the rib cage was smaller in the CKO embryos. The sternal bands failed to fuse completely resulting in a total sternal fissure in 12% (Fig 5A), or failed to fuse at the anterior portion of the sternum resulting in a partial sternal fissure in 24% of the CKO embryos (Fig 5B; Table 2). In addition, fewer ribs were detected in these CKO embryos, and some of the ribs did not attach to the vertebrae (Fig 6 and data not shown). The other 40% of the CKO embryos displayed a non-fusion at the caudal portion of the sternum leaving a perforation of the sternal body, and a lack of the manubrium (Fig 5C). In the rest of the CKO embryos, the sternum appeared relatively normal except it was shorter and the manubrium was hypoplastic (Fig 5D).

**Table 2:** Rib cage anomalies of *Pdgfra* CKO embryos of different Tm groups.

Rib cage defects	E9.5	E10.5
Total sternal fissure; missing ribs; some ribs were not attached to the vertebrae	12%	0%
Partial sternal fissure; missing ribs; some ribs were not attached to the vertebrae	24%	0%
Non-fusion of the caudal portion of the sternum; perforations of the sternal body; absence of manubrium	40%	8.3%
Hypoplasia of manubrium	24%	83%

% of the CKO embryos showing different rib cage anomalies was shown

Around 65% (31 out of 48 CKO embryos) of the CKO embryos developed spina bifida, a birth defect where there was incomplete closure of the vertebrae and membranes around the spinal cord (Fig 5F) (Table 1). Skeletal staining revealed that the vertebral arches of the CKO vertebrae failed to form the convex structure as observed in the control littermate. Transverse section of CKO embryos indicated that the vertebral arches failed to grow to cover the dorsal side of the spinal cord, and the spinal cord was covered by a thin membrane only (arrowhead; Fig 6a). In control littermates, neural arches grew and connected with cartilage at the dorsal side covering the spinal cord.

All the E18.5 CKO embryos of the E9.5 Tm group showed the phenotype of omphalocele, in which the intestines and liver remained outside of the abdomen in a sac (arrows; Fig 3; Table 1). Transverse section of E18.5 CKO embryos revealed that the body wall musculature was not developed properly, in that muscle could only be localized from the dorsal to the ventral lateral body wall of the trunk (Fig 6b and 6c). No muscle could be detected at the ventral body wall of the CKO embryos, and the ventral body wall was much thinner as compared to that of the control littermates.

### Conditional knockout of *Pdgfra* in *Pdgfra*-expressing tissues at later embryonic day resulted in milder developmental anomalies

**Fig 7:**
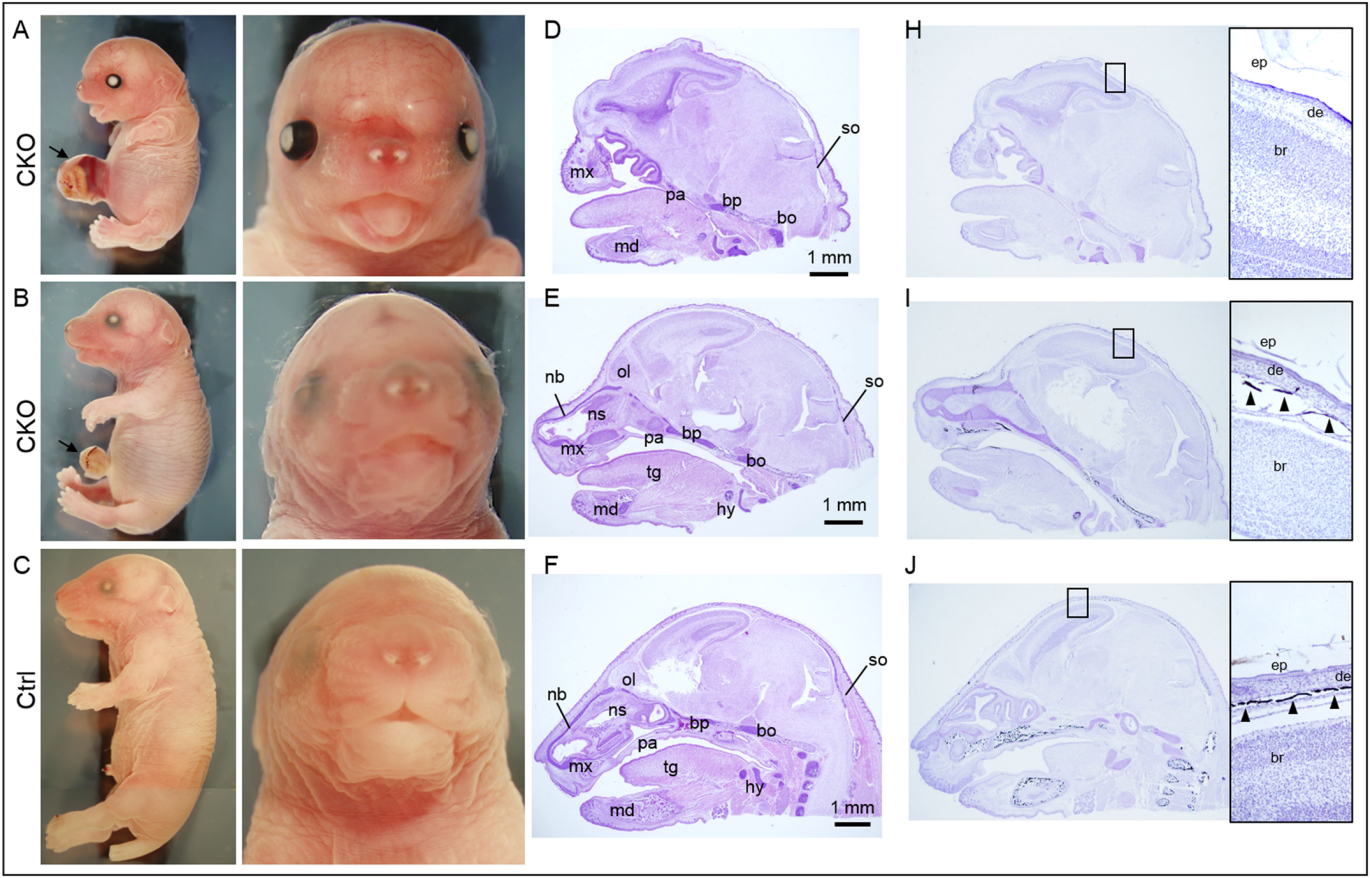
Conditional knockout of *Pdgfra* in *Pdgfra*-expressing tissues at E10.5 resulted in milder developmental anomalies. *Pdgfra*^*Cre/ERT*^;*Pdgfra*^*fl/+*^ mice were crossed with *Pdgfra*^*fl/fl*^, tamoxifen was given to pregnant females at E10.5 for two consecutive days. Conditional knockout (CKO, *Pdgfra*^*Cre/ERT*^;*Pdgfra*^*fl/fl*^) and control (Ctrl, *Pdgfra*^*fl/fl*^) embryos were collected at E18.5 for analysis. Morphological examinations of CKO embryos (A, B) and control littermate (C). Haematoxylin and eosin staining of sagittal sections of the head of CKO embryos (D, E) and control littermate (F). Von Kossa staining (black) of the sagittal sections of the head of CKO embryos (H, I) and control littermate (J). Highlighted regions were magnified and shown on the right, and arrowheads indicated the positive staining of bone. Arrows indicated omphalocele. Abbreviations: tg, tongue; md, mandible; mx, maxilla; pa, palatal shelf; bp, basisphenoid bone; bo, basioccipital bone; nb, nasal bone; ns, nasal septum; hy, hyoid bone; so, supraoccipital bone; ep, epidermis; de, dermis; br, brain; bo, bone.

Similar skeletal abnormalities could also be detected in the CKO embryos of the E10.5 Tm group, but in general, with lower incidence and milder severity of the abnormalities. No cleft face and cleft palate was detected in these CKO embryos, and only 8% of the CKO embryos showed shorter snout and a partial lack of the skull bone (Fig 7; Table 1). In addition, these CKO embryos also exhibited defective eyelids development like the CKO embryos of E9.5 Tm group. The rest of the CKO embryos (92%) showed normal snout length, and normal nasal cavity with the nasal bone and the nasal septum normally developed comparable to that of the normal control littermates. The skull bone was formed in these CKO embryos as revealed by Von Kossa staining of the cranial region (Fig 7D–7F). The neural crest cell-derived and cephalic mesoderm derived bones were also developed in the majority of the CKO embryos of the E10.5 Tm group. Milder skeletal abnormalities of the rib cage and the vertebral column were detected in the CKO embryos of the E10.5 Tm group (S4 Fig). Shorter sternum was detected in the CKO embryos, with the missing of the manubrium or manubrium hypoplasia in 8% and 92% of these embryos, respectively (Table 2). Spina bifida was only detected in 2 (17%) CKO embryos (Table 1). However, omphalocele was detected in all the CKO embryos (arrows, Fig 7; Table 1). The CKO embryo of the E11.5 Tm group only showed the phenotype of omphalocele, and the CKO embryo of the E13.5 Tm group did not develop any of the above mentioned anomalies (Table 1).

Taken all the above indicated that conditional knockout of *Pdgfra* in *Pdgfra*-expressing tissues in mouse embryos at different embryonic days (E9.5 and E10.5) resulted in multiple developmental anomalies of the frontonasal region, the cranium and the abdominal wall musculature (Table 1). Furthermore, the day at which tamoxifen was given had a major impact on the repertoire of the anomalies of the CKO embryos, and tamoxifen treatment at E9.5 resulted in the development of all the anomalies.

### Deletion of *Pdgfra* induced apoptosis

**Fig 8:**
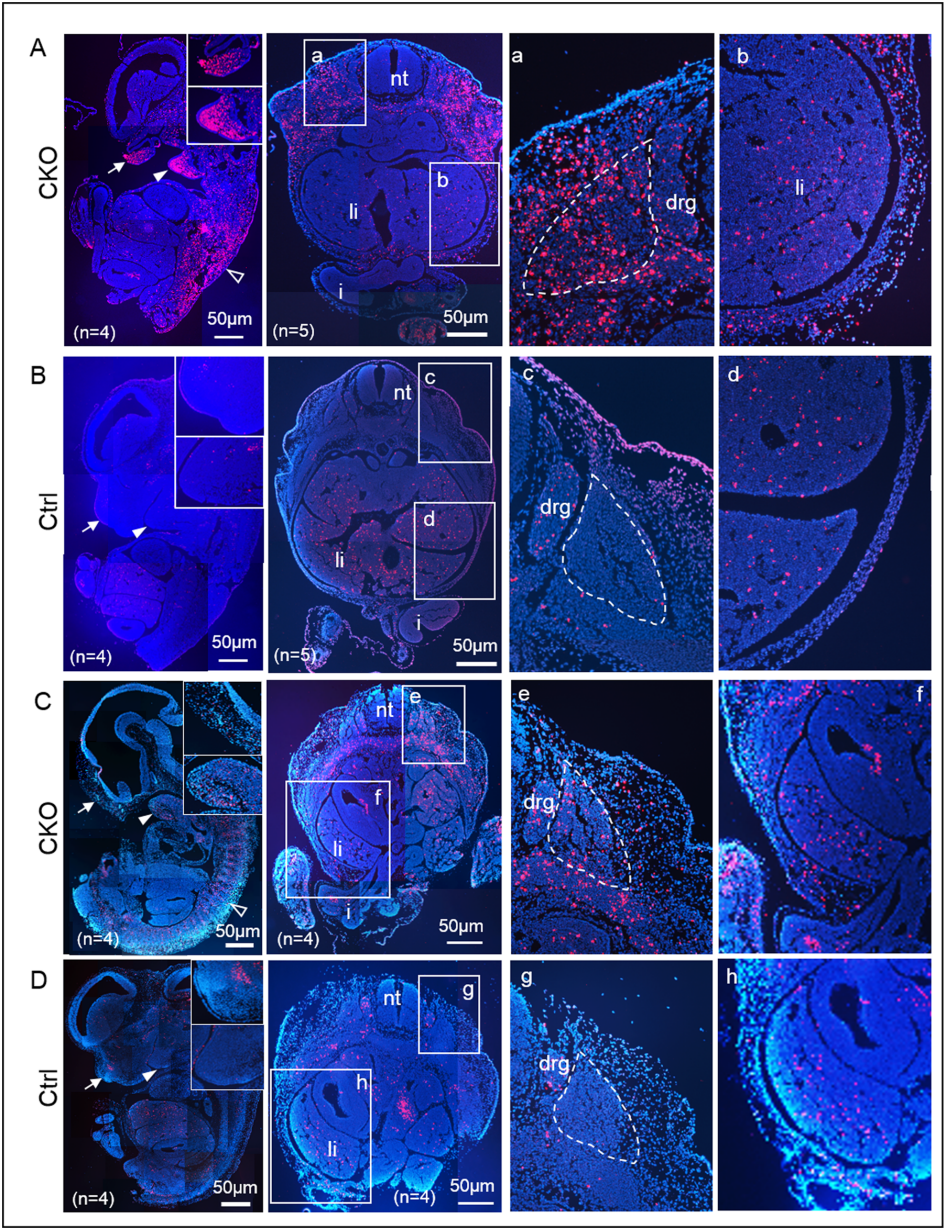
Deletion of *Pdgfra* induced apoptosis. Sagittal sections of CKO (*Pdgfra*^*Cre/ERT*^;*Pdgfra*^*fl/fl*^) and control (Ctrl, *Pdgfra*^*fl/fl*^) E12.5 embryos of E9.5 Tm group were examined by TUNEL assay (Red). Highlighted regions were magnified and shown on the right. Number of embryos analyzed for each group was indicated as “n”, and representative photo of each group was shown. Abbreviations: nt, neural tube; li, liver; i, intestine; drg, dorsal root ganglion.

To test if deletion of *Pdgfra* induced apoptosis of the *Pdgfra*-expressing tissues in embryos, CKO and control E12.5 embryos of E9.5 and E10.5 Tm groups were examined by TUNEL assay. Apoptotic signals were localized at the frontonasal region (arrow), the mandibular (arrowhead) processes, the somites, and at the developing dorsal body wall (Fig 8A–8D). Elevated cell death could also be localized at the region of the cephalic mesenchyme. However, the amount of apoptotic cells at these regions was markedly lower in the CKO embryos of E10.5 Tm group than that of E9.5 Tm group. TUNEL staining of the transverse section of the lumbar regions of the CKO E12.5 embryos localized abundant apoptotic cells at the somites (marked with dotted line), the mesoderm surrounding the somites as well as at the developing body wall. To quantitate the apoptosis of the CKO embryos and the control embryos, we determined the percentages of apoptotic cells (% apoptosis) of the frontonasal region, somite and abdominal wall of CKO and control embryos. Percentages of apoptosis were significantly higher in CKO embryos than their control littermates within the same Tm group (Table 3). However, % apoptosis of the frontonasal region, and the somite of the E12.5 CKO embryos of E10.5 Tm group were significantly lower than their respective regions of E9.5 Tm group. Interestingly, % apoptosis of the abdominal wall were similarly in CKO embryos of E9.5 and E10.5 Tm groups.

**Table 3:** Apoptosis of the frontonasal region, somite and abdominal wall in *Pdgfra* CKO embryos of different Tm groups.

Tm	E9.5		E10.5		E11.5*	
	Control (n = 4)	CKO (n = 4)	Control (n = 4)	CKO (n = 4)	Control (n = 6)	CKO (n = 5)
Region						
	% apoptosis		% apoptosis		% apoptosis	
Frontonasal	2±0.3%	33.8±4.2%	1.1±0.1%	10±0.4%	N.D.	N.D.
		*p* = 0.009^§^		*p* = 0.001^§^		
				*p* = 0.02^#^		
Somite	2.2±1.2%	31.1±1.1%	0.9±0.1%	9.4±1.1%	1.8±0.1%	2±0.2%
		*p* = 9.8E-05^§^		*p* = 0.007^§^		*p* = 0.1^§^
				*p* = 0.002^#^		
Abdominal wall	3.7±0.1%	16.1±2.1%	6±1.1%	17.2±0.2%	17.2±3.4%	39.2±0.8%
		*p* = 0.02^§^		*p* = 0.006^§^		*p* = 0.01^§^
				*p* = 0.58^#^		

^§^: *p* value of comparison between control (*Pdgfra*^*fl/fl*^) and CKO (*Pdgfra*^*Cre/ERT*^;*Pdgfra*^*fl/fl*^) embryos of the same Tm group;

^#^: *p* value of comparison between CKO (*Pdgfra*^*Cre/ERT*^;*Pdgfra*^*fl/fl*^) embryos of the Tm E9.5 and Tm E10.5 group.

*: control (*Pdgfra*^*fl/fl*^) and CKO (*Pdgfra*^*Cre/ERT*^;*Pdgfra*^*fl/fl*^) embryos were collected at E14.5. N.D., not determined.

To further investigate apoptosis of the somite and the abdominal wall in CKO embryos of Tm administration at later time point, we collected and examined apoptosis of the somite and body wall of the lumbar regions of the E13.5 control and CKO embryos of E11.5 Tm group by TUNEL staining on sections. Apoptotic cells in the somite of the CKO embryos were as few as that of the control embryos (S5 Fig; Table 3). In contrast, apoptotic cells were abundantly found in the body wall of the CKO embryos (S5 Fig), and % apoptosis in the body wall of CKO embryos was significantly higher than that of control embryos (Table 3).

## Discussion

The correct time and level of expressions of *Pdgfra* during embryonic development is crucial for proper development of endoderm, mesoderm derivatives. Current study showed that (i) conditional deletion of *Pdgfra* in *Pdgfra*-expressing tissues at different embryonic days resulted in developmental anomalies of the neural crest (NC) and somite derived structures, and (ii) the day at which *Pdgfra* was deleted had a major impact on the combination of the anomalies of the conditional *Pdgfra* knockout embryos.

The axial skeleton consists of the craniofacial skeleton, the rib cage, the sternum and the vertebral column, and is mainly derived from cranial NC and paraxial mesoderm (somites) [30–32]. Craniofacial skeleton is derived from two main sources of cells: the cranial NC and the cranial mesoderm (CM) [33–38]. The cranial NC contribute to the lower and the upper jaws, the snout, the frontal bones of the skull and the anterior skull base. The CM contributes to the bones of the neurocranium and the posterior skull base [37, 39–41]. The rib cage and the vertebrae are derived from somites.

Using the *Pdgfra*^*Cre/ERT*^; *Pdgfra*^*fl/+*^ mice and *Rosa26R* (*R26R*) [29], we showed that tamoxifen administration at E9.5 induced a robust X-gal expression to the regions whereas the endogenous *Pdgfra* was expressed, including the mesoderm of the cephalic region, the nasal process, the maxillary and the mandibular processes, and the developing somites. Crossing the *Pdgfra*^*Cre/ERT*^ [26] mice with *Pdgfra*^*fl/fl*^ [15], and giving tamoxifen at E9.5 resulted in defective development of cranial NC derived and CM derived cartilages and bones of the frontonasal region and the skull; abnormal development of the vertebrae, the rib cage, and the abdominal wall musculature. These affected structures of the *Pdgfra* CKO embryos are derivative of the cephalic mesoderm and cranial NC, mesoderm of the cephalic region, the medial and lateral nasal processes, the 1^st^ and the 2^nd^ brachial arches, and the developing somites in mice. The eyelids develop from both secondary mesenchyme and surface ectoderm, and cranial NC is the primary source of the mesenchymal component of the eyelids. Therefore, failure of eyelid development in the *Pdgfra* CKO embryos is likely due to the defective development of the mesenchymal derivatives of the cranial NC.

In E12.5 CKO embryos, apoptotic cells were abundantly localized at the frontonasal region, the mandibular process, the region of the cephalic mesenchyme, the developing somites, the mesoderm surrounding the somites as well as of the developing body wall. These data indicate that *Pdgfra* is important for the survival of mesoderm and its derivatives, NC, somite and its derivatives. Elevated apoptosis of these structures could lead to skeletal development defects in *Pdgfra* CKO embryos.

The body wall development is divided into the primary and the secondary body wall formation. The ectoderm and the lateral plate mesoderm elongate laterally and coalesces at the ventral midline around the umbilicus to form the primary abdominal wall enclosing the abdominal cavity. The myoblasts migrate out of the myotome into the primary body wall and differentiate to form the secondary body wall formation. The apoptosis of the developing body wall of the CKO embryos could lead to the defective secondary body wall development, and omphalocele.

NC-specific deletion of *Pdgfra* resulted in defects of craniofacial and aortic arch detects [15]. Defective development of lung alveolar mesenchyme, intestinal mesenchyme, oligodendrocytes, Leydig cells, and the diaphragm have also been described in the population of surviving *Pdgfra* null mutants [42–46]. We have not detected any defect of the aortic arch, the lung alveolar mesenchyme and the diaphragm in all the *Pdgfra* CKO embryos of the E9.5 Tm group (data not shown). Lack of lung developmental defect in the *Pdgfra* CKO embryos could be due to the ineffective deletion and of *Pdgfra* expression in the developing lung of CKO embryos of the E9.5 Tm group (S3 Fig).

In mouse, the cardiac NC emigrates from the NT at around E8, and colonizes the outflow tract at around E10.25 [47]. In general, Tm administration by gastric gavage of pregnant mice in mouse induced cre-mediated deletion of *floxed* allele at 18 to 24 hours post administration. Therefore, the *Pdgfra* gene was deleted in *Pdgfra* expressing tissues by E10 to E10.5 in CKO embryos, which has already passed the critical time point of NT emigration and outflow tract colonization of the cardiac NC. This could explain the lack of aortic arch defect in our CKO embryos.

Present study showed that apoptosis of the frontonasal region, mandibular process, somite and abdominal body wall of CKO embryos was greatly affected by the day at which the *Pdgfra* was deleted. *Pdgfra* deletion at earlier embryonic stages caused excessive apoptosis of these regions, while *Pdgfra* deletion at later stage resulted in much reduced apoptosis and only in a subset of these regions. The repertoire of the anomalies of the CKO embryos correlated with the degrees of apoptosis of these different regions.

In conclusion, conditional knockout of *Pdgfra* in *Pdgfra*-expressing tissues in mouse embryos at different embryonic days (E9.5 and E10.5) resulted in multiple developmental anomalies of the frontonasal region, the cranium and the abdominal wall musculature. Furthermore, the day at which the *Pdgfra* is deleted influences the repertoire of the anomalies of the CKO embryos.

Orofacial cleft (includes cleft lip, cleft palate, and both together), spina bifida (includes spina bifida occulta, meningocele, and myelomeningocele) and omphalocele are among the commonest skeletal and abdominal wall defects of newborns. The remarkable resemblance of our conditional *Pdgfra* CKO embryos to theses human congenital anomalies, suggesting that dysregulation of the time and level of the expression of *PDGFRA* could cause these anomalies in human. Future work should aim at defining (a) the regulatory elements for the transcriptional regulation of the human *PDGFRA* during embryonic development, and (b) if mutations / sequence variations of these regulatory elements cause these anomalies.

## Supporting information

S1 Fig PCR genotyping of mice for *Cre*, wild-type and floxed *Pdgfra* and *Sry*. (A) The wild-type *Pdgfra*, the floxed *Pdgfra* locus and the *Pdgfra*^*Cre/ERT*^ locus were shown. Black boxes represent exons; ATG indicates the start of translation. The floxed allele contains the Neo cassette (gray box) and the two *loxP* sites (black arrowheads). Primers for PCR amplification are showed as arrows. (B) Agarose gel electrophoresis of PCR products of genomic DNAs. PCR product respective of *Pdgfra*^*Cre/ERT*^, wild-type *Pdgfra*, and the floxed *Pdgfra* allele were indicated with arrows. (C) The wild-type *Pdgfra* locus was shown, and the locations of the forward and reverse primers (arrows) and the TaqMan probe (black bar) for the determination of the copy number of *Pdgfra* allele were shown. Locations of the restriction endonucleases were indicated.
(TIF)

S2 Fig Conditional deletion of *Pdgfra* at E7.5 resulted in phenotypic abnormalities and embryonic lethality in mutant embryos. E14.5 mutant embryos (CKO, *Pdgfra*^*Cre/ERT*^;*Pdgfra*^*fl/fl*^) were generally smaller, and displayed cleft face, bleeding, subepidermal bleb (arrowhead). Control littermate (Ctrl, *Pdgfra*^*fl/fl*^) was shown for comparison.
(TIF)

S3 Fig Immuno-histochemistry for PDGFRA in CKO mutants. Conditional knockout (CKO, *Pdgfra*^*Cre/ERT*^;*Pdgfra*^*fl/fl*^) and control (Ctrl, *Pdgfra*^*fl/fl*^) embryos were collected at E12.5 of E9.5 Tm group for immunostaining for PDGFRA. Upper panel, immuno-reactivity for PDGFRA (brown) were localized in the mesenchyme tissues at the fourth ventricle (A), the developing eye (B), and the nasal region (C, D) of the control embryos. Immuno-reactivity for PDGFRA was absent in the mesenchyme tissues of these regions (E, F, G) of the CKO embryos. Lower panel, immuno-reactivity for PDGFRA (brown) were localized in the somite (A) and the developing body wall (B), but not in the CKO embryos (C, D). However, PDGFRA protein was localized at the developing lung (arrowhead) in both CKO and control embryos.
(TIF)

S4 Fig Rib cage anomalies of conditional *Pdgfra* knockout embryos. Skeletal staining of the rib cages of conditional *Pdgfra* knockout (CKO, *Pdgfra*^*Cre/ERT*^;*Pdgfra*^*fl/fl*^) (A-B) and control (Ctrl, *Pdgfra*^*fl/fl*^) (C) embryos of E10.5 Tm group were shown for comparison. The ribs were numbered and arrows indicated the respective location of the manubrium. Abbreviations: m, manubrium; st, sternum; xp, xiphoid process.
(TIF)

S5 Fig Deletion of *Pdgfra* induced apoptosis. Sagittal sections of CKO (A, *Pdgfra*^*Cre/ERT*^;*Pdgfra*^*fl/fl*^) and control (B, Ctrl, *Pdgfra*^*fl/fl*^) E14.5 embryos of E11.5 Tm group were examined by TUNEL assay (Red). Highlighted regions were magnified and shown on the right. Number of embryos analyzed for each group was indicated as “n”, and representative photo of each group was shown. Abbreviations: nt, neural tube; li, liver; i, intestine.
(TIF)

S1 Table Primers and PCR conditions for the detection of the Cre, wild-type and floxed Pdgfra and Sry. (DOCX)
