# Supplementary figures and images for: Stage specific requirement of platelet-derived growth factor receptor-α in embryonic development
Source: PLoS One. 2017 Sep 21;12(9):e0184473. doi: 10.1371/journal.pone.0184473 (PMC5608218; doi:10.1371/journal.pone.0184473)

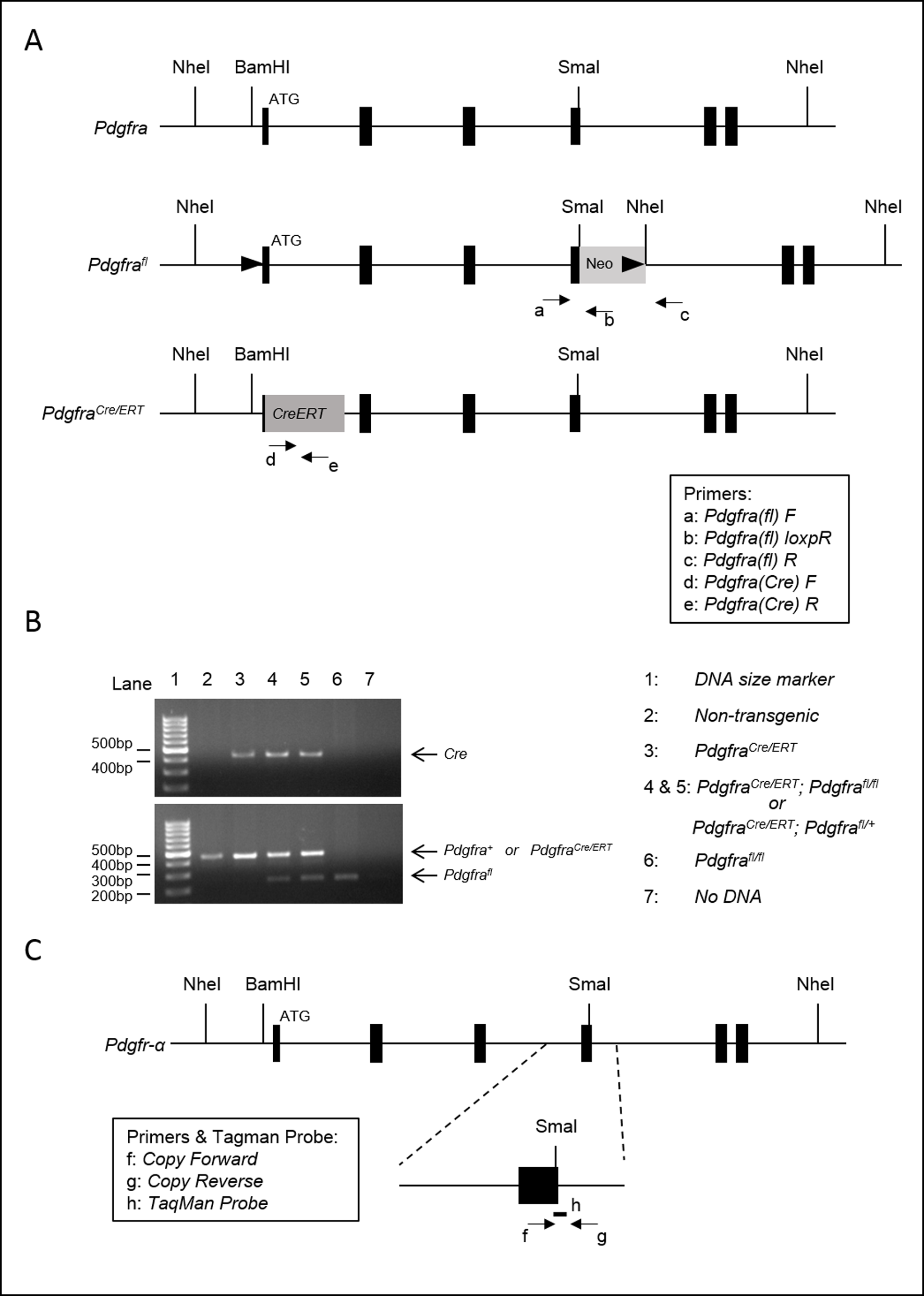

Supplement: S1 Fig — (A) The wild-type Pdgfra, the floxed Pdgfra locus and the PdgfraCre/ERT locus were shown. Black boxes represent exons; ATG indicates the start of translation. The floxed allele contains the Neo cassette (gray box) and the two loxP sites (black arrowheads). Primers for PCR amplification are showed as arrows. (B) Agarose gel electrophoresis of PCR products of genomic DNAs. PCR product respective of PdgfraCre/ERT, wild-type Pdgfra, and the floxed Pdgfra allele were indicated with arrows. (C) The wild-type Pdgfra locus was shown, and the locations of the forward and reverse primers (arrows) and the TaqMan probe (black bar) for the determination of the copy number of Pdgfra allele were shown. Locations of the restriction endonucleases were indicated. (TIF) [file pone.0184473.s001.tif]

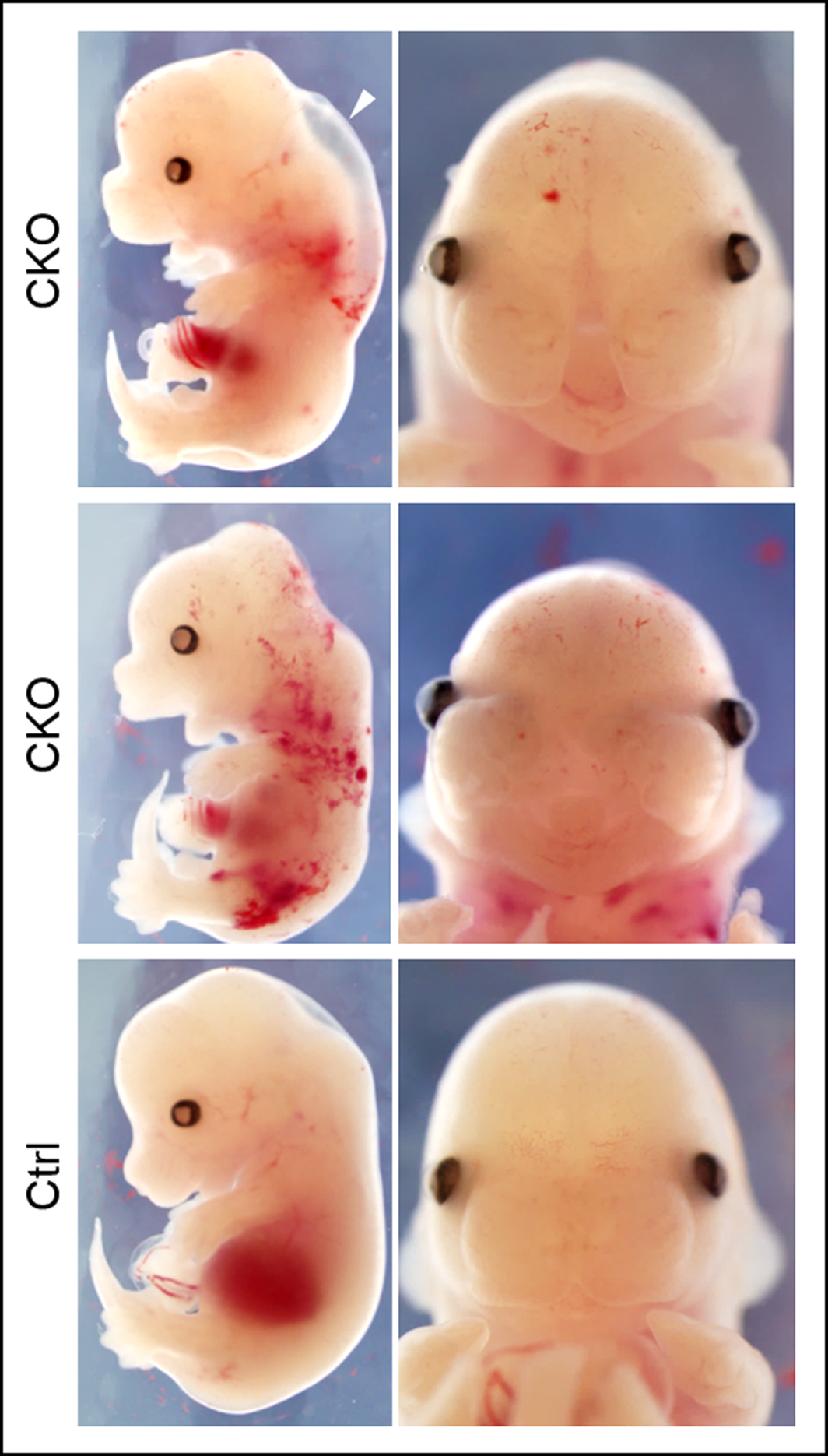

Supplement: S2 Fig — E14.5 mutant embryos (CKO, PdgfraCre/ERT;Pdgfrafl/fl) were generally smaller, and displayed cleft face, bleeding, subepidermal bleb (arrowhead). Control littermate (Ctrl, Pdgfrafl/fl) was shown for comparison. (TIF) [file pone.0184473.s002.tif]

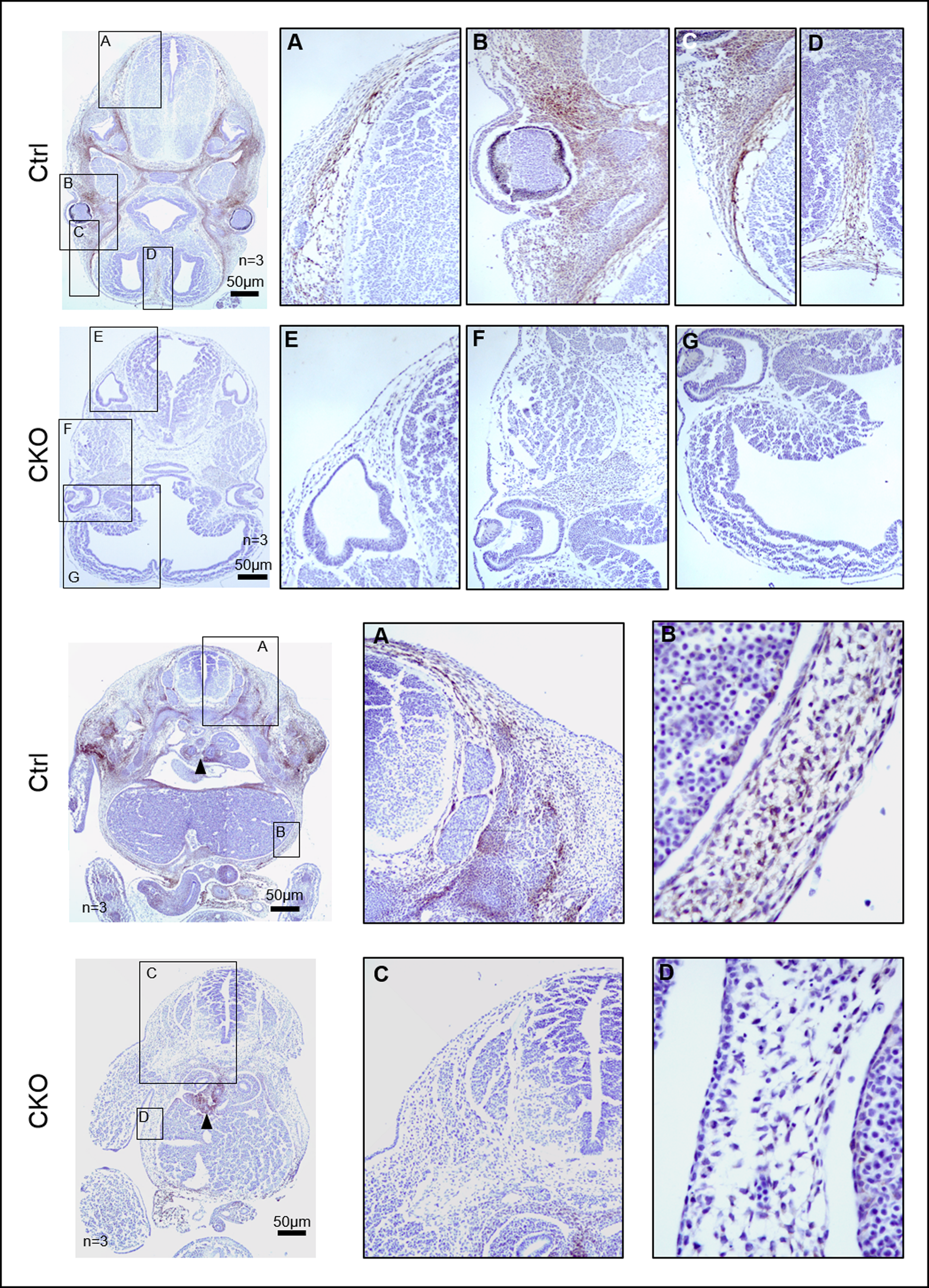

Supplement: S3 Fig — Conditional knockout (CKO, PdgfraCre/ERT;Pdgfrafl/fl) and control (Ctrl, Pdgfrafl/fl) embryos were collected at E12.5 of E9.5 Tm group for immunostaining for PDGFRA. Upper panel, immuno-reactivity for PDGFRA (brown) were localized in the mesenchyme tissues at the fourth ventricle (A), the developing eye (B), and the nasal region (C, D) of the control embryos. Immuno-reactivity for PDGFRA was absent in the mesenchyme tissues of these regions (E, F, G) of the CKO embryos. Lower panel, immuno-reactivity for PDGFRA (brown) were localized in the somite (A) and the developing body wall (B), but not in the CKO embryos (C, D). However, PDGFRA protein was localized at the developing lung (arrowhead) in both CKO and control embryos. (TIF) [file pone.0184473.s003.tif]

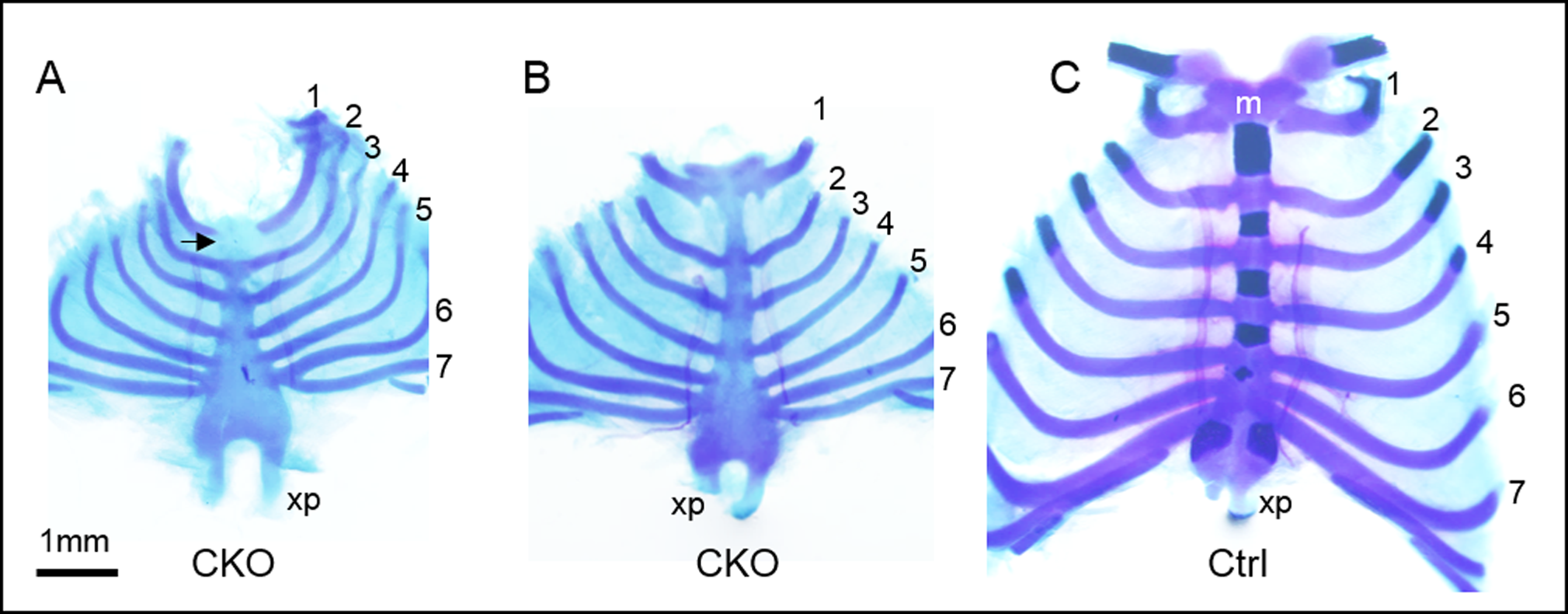

Supplement: S4 Fig — Skeletal staining of the rib cages of conditional Pdgfra knockout (CKO, PdgfraCre/ERT;Pdgfrafl/fl) (A-B) and control (Ctrl, Pdgfrafl/fl) (C) embryos of E10.5 Tm group were shown for comparison. The ribs were numbered and arrows indicated the respective location of the manubrium. Abbreviations: m, manubrium; st, sternum; xp, xiphoid process. (TIF) [file pone.0184473.s004.tif]

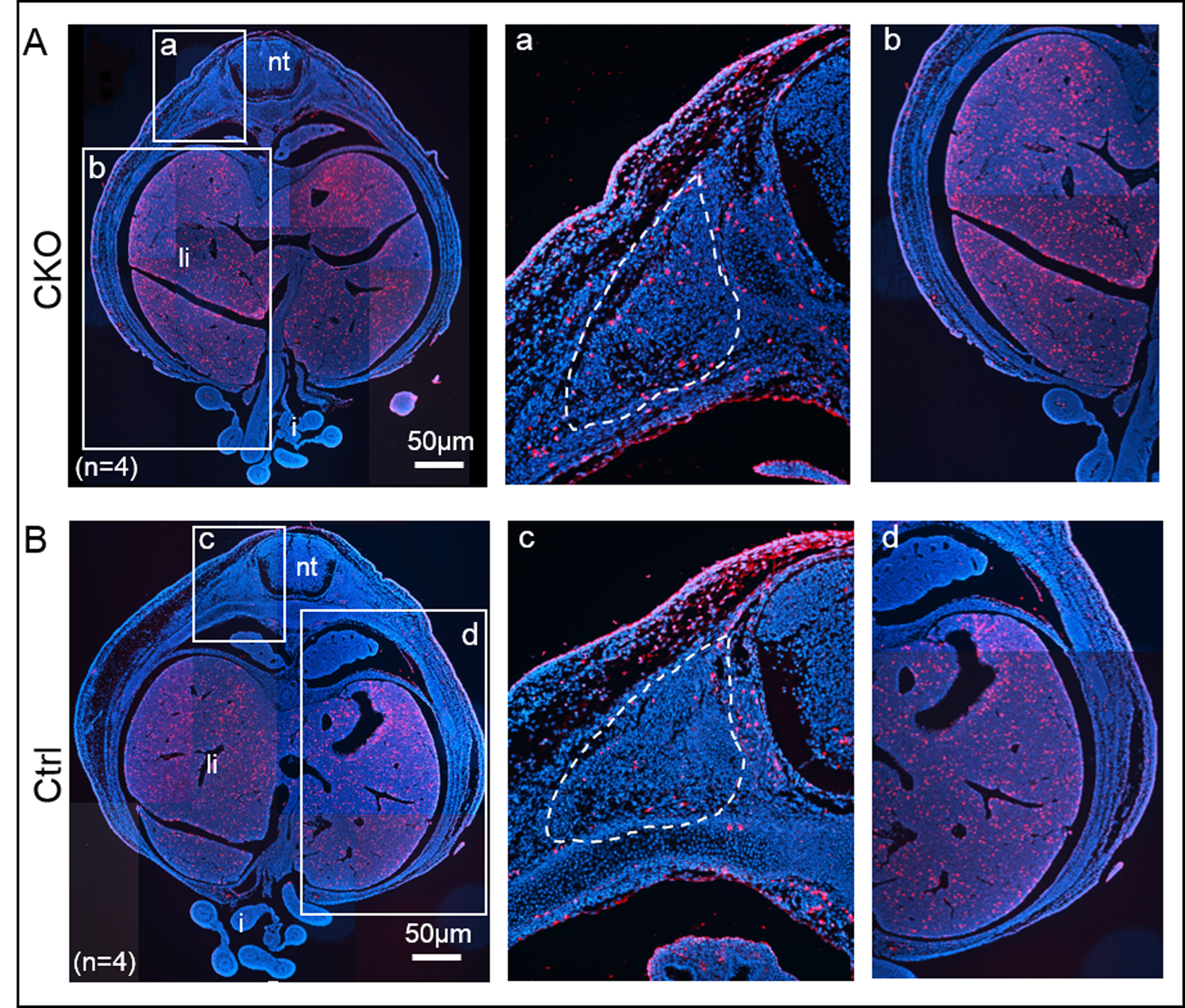

Supplement: S5 Fig — Sagittal sections of CKO (A, PdgfraCre/ERT;Pdgfrafl/fl) and control (B, Ctrl, Pdgfrafl/fl) E14.5 embryos of E11.5 Tm group were examined by TUNEL assay (Red). Highlighted regions were magnified and shown on the right. Number of embryos analyzed for each group was indicated as “n”, and representative photo of each group was shown. Abbreviations: nt, neural tube; li, liver; i, intestine. (TIF) [file pone.0184473.s005.tif]
