# Supplementary material for: Stage specific requirement of platelet-derived growth factor receptor-α in embryonic development
Source: PLoS One. 2017 Sep 21;12(9):e0184473. doi: 10.1371/journal.pone.0184473 (PMC5608218; doi:10.1371/journal.pone.0184473)
Supplement: S1 Table — (DOCX) [file pone.0184473.s006.docx]

S1 Table: Primers and PCR conditions for the detection of the *Cre*, wild-type and floxed *Pdgfra* and *Sry*.

| Allele | Primers | Sequence | PCR conditions | PCR products (bp) |
| --- | --- | --- | --- | --- |
| *Pdgfra cre* | *Pdgfra(Cre) F* | 5’-TCA GCC TTA AGC TGG GAC AT-3’ | 94°C, 5 min; 10 cycles: 94°C, 20 sec; 65°C, 20 sec; 68°C, 15 sec;  28 cycles: 94° C, 20 sec; 60° C, 20 sec; 72° C, 20 sec; 72°C, 10 min. | 492 |
|  | *Pdgfra(Cre) R* | 5’-ATG TTT AGC TGG CCC AAA TG-3’ |  |  |
| *Pdgfra flox* | *Pdgfra(fl) F* | 5’-GCT TTT GCC TCC ATT ACA CTG G A AC-3’ | 94°C, 5 min; 35 cycles: 94°C, 30 sec; 65°C, 60 sec; 72°C, 60 sec; 72°C, 10 min. | Floxed *Pdgfr-α*: 242  wildtype *Pdgfr-α*: 451 |
|  | *Pdgfra(fl) R* | 5’-CCC TTG TGG TCA TGC CA-3’ |  |  |
|  | *Pdgfra(fl) loxpR* | 5’-ACG AAG TTA TTA GGT CCC TCG AC-3’ |  |  |
| *sry* | *sry F* | 5’-AGA TCT TGA TTT TTA GTG TTC-3’ | 94°C, 5 min; 35 cycles: 94°C, 30 sec; 58°C, 45 sec; 72°C, 45 sec; 72°C, 10 min. | Male 467 |
|  | *sry R* | 5’-TGC AGC TCT ACT CCA GTC TTG-3’ |  |  |
| *Pdgfra* | *Copy Forward* | 5’-TTG ACC TGC AGT GGA CTT ACC-3’ | 95 °C, 1 min; 40 cycles: 95 °C, 15 sec; 60 °C, 1 min; 60 °C, 1 min. | N.A. |
|  | *Copy Reverse* | 5’-GGT GTG TAG AGT TAC AAG TCC TGT T-3’ |  |  |
|  | *TaqMan Probe* | 5’-ACC CCG GGA TAA GGA GCT CA-3’ |  |  |

Details of primers and TaqMan probe, PCR conditions and product sizes for each allele were shown. N.A.: Not Applicable
